# Evaluation of Motor Primitive-Based Adaptive Control for Lower Limb Exoskeletons

**DOI:** 10.3389/frobt.2020.575217

**Published:** 2020-12-16

**Authors:** Polyana F. Nunes, Icaro Ostan, Adriano A. G. Siqueira

**Affiliations:** Rehabilitation Robotics Laboratory, Department of Mechanical Engineering, São Carlos School of Engineering, University of São Paulo, São Carlos, Brazil

**Keywords:** rehabilitation robotics, motor primitives, exoskeleton, lower limbs, biomechatronics

## Abstract

In order to assist after-stroke individuals to rehabilitate their movements, research centers have developed lower limbs exoskeletons and control strategies for them. Robot-assisted therapy can help not only by providing support, accuracy, and precision while performing exercises, but also by being able to adapt to different patient needs, according to their impairments. As a consequence, different control strategies have been employed and evaluated, although with limited effectiveness. This work presents a bio-inspired controller, based on the concept of motor primitives. The proposed approach was evaluated on a lower limbs exoskeleton, in which the knee joint was driven by a series elastic actuator. First, to extract the motor primitives, the user torques were estimated by means of a generalized momentum-based disturbance observer combined with an extended Kalman filter. These data were provided to the control algorithm, which, at every swing phase, assisted the subject to perform the desired movement, based on the analysis of his previous step. Tests are performed in order to evaluate the controller performance for a subject walking actively, passively, and at a combination of these two conditions. Results suggest that the robot assistance is capable of compensating the motor primitive weight deficiency when the subject exerts less torque than expected. Furthermore, though only the knee joint was actuated, the motor primitive weights with respect to the hip joint were influenced by the robot torque applied at the knee. The robot also generated torque to compensate for eventual asynchronous movements of the subject, and adapted to a change in the gait characteristics within three to four steps.

## 1. Introduction

According to the World Health Organization (WHO), population aging is a fact and by 2050 the number of people aged 60 and over will reach 2 billion (World Health Organization, 2015) representing one fifth of the planet's population (Castles et al., 2013). The incidence of gait disorders can reach a prevalence of 35 and 60% in people over 70 and 80 years old, respectively, and the most common disorder among these people is stroke. Worldwide, stroke is the second leading cause of death and the third biggest cause of long-term disability, with approximately 33 million surviving individuals (Lozano et al., 2012; Murray et al., 2015). Among the various anomalies caused by it, stroke can originate severe sequelae to the neural areas that control upper and lower limbs movements, since it leads to a lack or an excess of blood supply in the brain, which affects about 50% of survivors (Mackay et al., 2004). As a consequence, most post-stroke individuals do not have a balance and support phase defined as in the gait of a normal individual, leading to a greater risk of falls, as movements are uncontrolled, and balance and proprioception are impaired (Sommerfeld et al., 2004).

To improve the quality of life of these individuals is only possible because the cerebral cortex is formed by a set of interconnected neuronal cells, which, in response to changes in the environment, are able to adapt. This adaptation occurs due to the fundamental property of the nervous tissues that form the basis of learning (or relearning), called neuroplasticity. This depends exclusively on repetitive experiences that will contribute to motor recovery after stroke or any other injury to the central nervous system (CNS) (Wieloch and Nikolich, 2006; Pekna et al., 2012). CNS neuroplasticity contributes to the development of motor primitives (muscle activity pattern), which combine with flexibility to produce motor behaviors (d'Avella et al., 2003; Ting and McKay, 2007; Bizzi et al., 2008; Tresch and Jarc, 2009; Bizzi and Cheung, 2013).

In view of the difficulties faced by these individuals after the stroke and with the objective of recovering lost or impaired movements, thus improving the quality of life of these people, several studies on rehabilitation have been carried out by researchers in the field of robotics, which aim to develop new strategies to recover gait symmetry with the aid of adaptive robots (Krebs et al., 2008; Contreras-Vidal et al., 2016; Zadravec et al., 2017). In a recent study with post-stroke individuals using a lower limbs exoskeleton, it was possible to observe an increase in lateral symmetry of muscle synergies during walking and a significant evolution in gait kinematics after 3 weeks of training (Tan et al., 2018).

In the rehabilitation process, one of the most important tasks has been to accurately determine the levels of assistance that will act on human joints during gait and a recent study published by Fricke et al. (2020) showed apparent advantages of automatic adjustment over manual adjustment performed by therapists during clinical practice. Therefore, it is possible to develop an adaptive control strategy based on the knowledge of the user's kinetic characteristics, so that the exoskeleton can react intuitively to the movement intended by the user, providing coherent, collaborative and effective assistance, which has been the objective of several recent researches (Diaz et al., 2011; Yan et al., 2015; Alibeji et al., 2018; Bayon et al., 2018; Maggioni et al., 2018).

**Figure 1:**
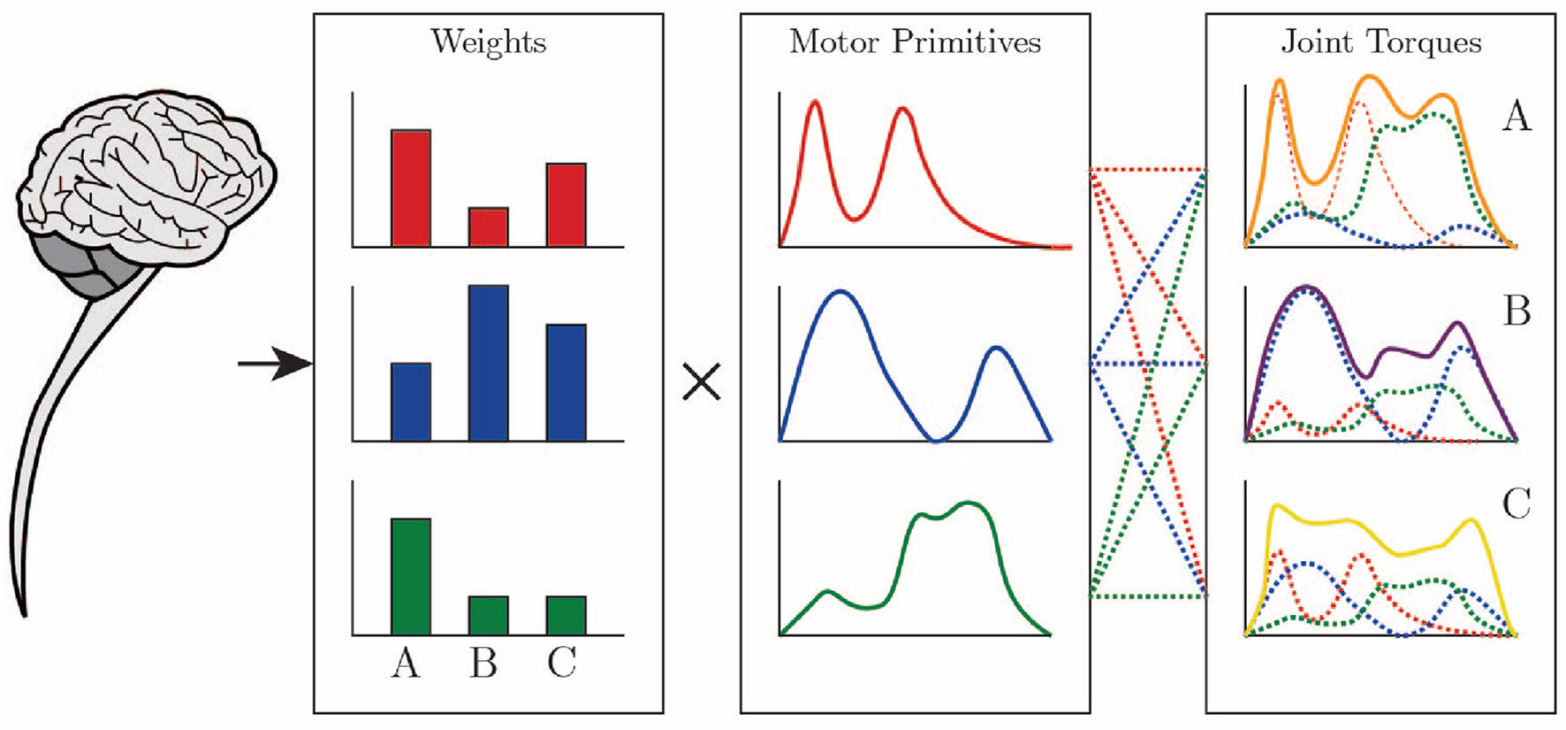
Joint torques are constructed through a weighted sum of motor primitives by the CNS. Drawing based on Ting et al. (2015).

The use of motor primitives, as depicted in Figure 1, can significantly reduce the computational load during motor control over the CNS, since, with a basic set of primitives, it is possible to reconstruct different conditions and tasks related to a movement, just by defining when and how much to recruit from each primitive within the movement segment of each individual (Ruckert and d'Avella, 2013). The movement of the musculoskeletal system is influenced by the neural and biomechanical systems of the body and their interaction with the environment. Controlling this system is a complex task due to the abundance of degrees of freedom, with which the central nervous system is not in a position to deal initially (Bernstein, 1967).

Neuroplasticity is the neurobiological basis of the ability to adapt and learn in a manner dependent on experience, through repetitions (Wieloch and Nikolich, 2006), generating new movement patterns and new motor primitives (Bizzi et al., 2008). Such primitives have been associated with biomechanical functions necessary for walking, producing in response a specific motor output with specific patterns of muscle activity that restore previously lost functions. There is no correct or ideal motor pattern for each type of movement. The ability to choose between different solutions is implicit in the adaptability and robustness of biological systems, that is, different primitives can perform, in an equivalent way, the same type of motor task (Netune et al., 2009; Chvatal et al., 2011; Allen and Netuno, 2012).

The kinematic primitives were called sub-movements in studies carried out by Rohrer et al. (2002) about changes in the smoothness of movements in the recovery of post-stroke patients. In the same study, robotic therapy devices were used to analyze five measures of smoothness of hemiparetic movement in 31 patients recovering from stroke. The kinematic and dynamic adaptability to variations in precision, strength, and speed of movement under different conditions were analyzed in Grinyagin et al. (2005). In this last study, the kinematic variables were used to calculate the inverse dynamics, in order to obtain the torques between the articulations. To quantify the contributions of the joints individually in dynamic and kinematic primitives, the joint angles and torques were calculated by Principal Component Analysis (PCA).

In Garate et al. (2016), the concept of assistance based on primitives using an assistive exoskeleton was explored using two different approaches. In the first approach, to produce the desired assistive torque profiles, a combination of the primitives with the weights was made, and in the second approach, the motor primitives are identified to be inserted as neural stimuli in a virtual model of the musculoskeletal system. Kinematic variables of hip, knee, and ankle joint positions were used in the processing of data obtained with Inertial Measurement Units (IMU) in Nunes et al. (2018). Using the inverse dynamics of OpenSim, kinetic data (hip, knee, and ankle torques) were calculated to assess the influence of the exoskeleton structure on kinetic and muscle characteristics, using the relationships between motor primitives and their respective weights for the different conditions of use of the exoskeleton.

Herein, a bio-inspired controller for locomotion of wearable robots based on the concept of motor primitives is presented. The robot torque is calculated considering the particular kinetic motor primitives of a user, generating a force at the user's joint. A modular lower limb exoskeleton, presented in dos Santos et al. (2017a), was used to evaluate the control proposal. The device was configured to provide partial support during gait, in knee flexion and extension, applying precise torques calculated automatically by the control algorithm. As the torque of the exoskeleton is based on the primitives, it will always assist in order to compensate for the primitives with less weight, i.e., the ones which reflect some sort of lack of strength at the joint. The robot torque was applied to healthy subjects wearing the right leg of lower limbs exoskeleton, while walking on a treadmill. The healthy subjects walked actively, following the treadmill speed, and, in order to simulate a gait with a lack of torque, the subjects were later instructed to walk at a slower pace. The user's joint torques are provided by an estimation algorithm based on a generalized momentum-based disturbance observer (DOB) approach along with an extended Kalman filter, as described in dos Santos and Siqueira (2019). The hip and ankle joints are not actuated whereas the knee joint, which comprises a rotary series elastic actuator (rSEA), is actuated.

This work is organized as follows: the next section provides more details with respect to the equipment and control algorithm. In sequel, section 3 refers to the test procedure, which is followed by the test results in section 4. Section 5 discusses the results and is followed by the conclusion.

## 2. Methods

### 2.1. Lower Limbs Exoskeleton

**Figure 2:**
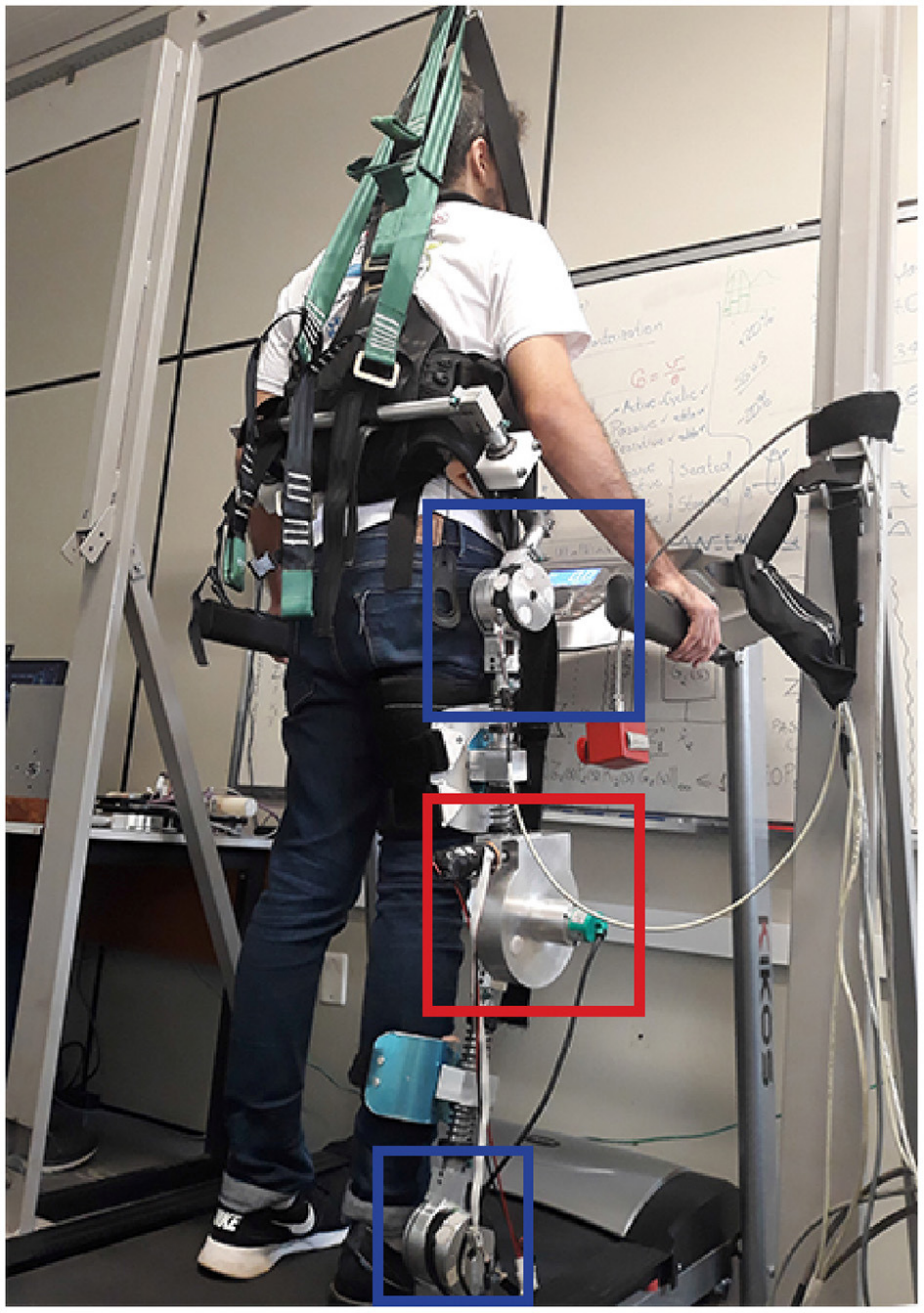
Test setup with healthy subject wearing the right leg of the lower limbs exoskeleton ExoTao. The red box delimits the rotary SEA at the knee joint. The blue boxes depict the hip and ankle joints, which are not actuated. The robot is suspended by a mechanical structure in order to compensate for part of its weight, which leads to a more comfortable walk on the treadmill.

The exoskeleton utilized in this work is the ExoTao (dos Santos et al., 2019). This robotic device comprises six rotary, one-degree-of-freedom joints, which can track the hip, knee, and ankle joint movement on the sagittal plane, with the aid of magnetic encoders. For the time being, only the right leg of the robot is considered. The only active joint is the knee, whereas the hip and the ankle work passively. The knee joint consists of a module with a rotary Series Elastic Actuator (SEA), as presented in dos Santos et al. (2017a), which can be configured to work in impedance and force control modes. Its motor is connected to an EPOS controller board, which communicates with the control algorithm running on a desktop computer via Controller Area Network (CAN) ports. In order to prevent the subject from slipping while walking, and to compensate for part of weight of the exoskeleton, a mechanical structure was installed as depicted in Figure 2.

The joints were designed to attend different ranges of motion with respect to different tasks. However, they can be limited by adjustable end-stops to prevent hyperextension of the joints. The exoskeleton contains Velcro®straps and telescopic links, which make it adjustable to different body heights from 1.60 to 1.90 m, in order to align the exoskeleton and the subject joints (dos Santos et al., 2017b).

### 2.2. Reference Values Extraction

To extract the primitives and analyze the data, aiming at the reduction of dimensionality and elimination of unnecessary characteristics, the Principal Component Analysis (Person, 1901) was employed. The PCA was chosen because it proved to be an accurate estimate to obtain a linear combination of primitives and their respective weights, in order to minimize the difference between the original and reconstructed signals (Nunes et al., 2020).

In the literature, primitives are usually calculated as the sum of the product between them, **p** = [**p_1_**, **p_2_**, …, **p_n_**] where **p** ∈ ℝ^*t*×*n*^, and their respective weights, **w** = [**w_1_**, **w_2_**, …, **w_n_**] where **w** ∈ ℝ^*n*×*n*^ as in Equation (1) (Cheung et al., 2009; Bizzi and Cheung, 2013; Berger and d'Avella, 2014; Roh et al., 2015). This calculation extracts a linear combination between primitives and their corresponding weights to minimize the difference between the original and reconstructed signals, which, in this case, are torque profiles **τ** ∈ ℝ^*n*×*t*^:

(1) $$\begin{array}{l} \tau =\text{p}\cdot \text{w} \end{array}$$

The motor primitives of four healthy subjects (1 female, 3 male, 30 ± 6 years, 73 ± 6 kg, 1.78 ± 0.04 m) were extracted in order to be further used by the control algorithm as reference. To extract these motor primitives, the subjects walked on a treadmill using the right leg of the exoskeleton for 2 min at 1.0 km/h. The knee joint of the robot was adjusted to impedance mode, thus, the knee of the subjects followed a desired pre-recorded trajectory, according to the control law:

(2) $$\begin{array}{l} \tau_{r,imp}=K_{v}\left(\theta^{d}-\theta \right), \end{array}$$

where *K*_*v*_ is the virtual stiffness of the impedance controller (set to 20 N.m/rad), τ_*r,imp*_ is the torque of the robot, while θ^*d*^ and θ are the desired trajectory and the knee joint measurement, respectively. An inner Proportional-Integral (PI) torque controller ensures that the robot torque is exerted at the joint.

The torque exerted by the subject's hip and ankle joints is estimated by means of an estimation algorithm that employs a generalized momentum-based disturbance observer (DOB) approach along with an Extended Kalman Filter. The Kalman Filter returns a time-varying gain at the end of every iteration, which ensures robustness with regards to the time-varying characteristics of the patient-exoskeleton model, as described by dos Santos and Siqueira (2019). The knee torque is obtained directly from the SEA.

This procedure yields the motor primitives, **p_h_**, and their respective weights, **w_h_**, with respect to each joint. Further auxiliary data that is also considered by the control algorithm are also generated, such as maximum primitive torque, τ_*h,max*_, and constant torque offset values, **τ**_*h,dc*_. The subscript **h** denotes the word healthy.

### 2.3. Control Algorithm

In summary, at the end of every step, the control algorithm computes the subject's motor primitive weights, based on the primitive torques extracted beforehand and on the joint torque measurements during the last swing phase. The subject's weights that are below the reference value are identified and, during the next swing phase, the robot actuates in order to compensate for the lack of weight. The following paragraphs explain in details the calculations performed by the control algorithm.

The algorithm is provided with the data from the procedure described in section 2.2, i.e., the reference or healthy values. After the right leg of the subject has performed the *k*^*th*^ swing, his torque curves, **τ**_*pat*_, with respect to the hip, knee, and ankle joints are obtained by the estimation algorithm and the SEA. These torque curves are treated before being employed to compute the subject motor primitive weights. First, their constant offset value, **τ**_*pat,dc*_, is subtracted. Afterward, they are divided by the extreme torque value obtained from the reference, τ_*max,h*_, resulting in a normalized torque profile, **τ**_*pat,n*_. This extreme value is the maximum value within each torque curve, and among torque curves. Due to the characteristic of the torque curves during the swing phase, it is known beforehand that this value is positive and related to the hip joint. However, in case other gait phases or tasks are analyzed, the algorithm handles the circumstance in which this value is a global minimum instead.

The aforementioned manipulations are summarized by the following equation:

(3) $$\begin{array}{l} \tau_{pat,n}^{\;k}=\left(\frac{1}{\tau_{h,max}}\right)\left(\tau_{pat}^{\;k}-\tau_{pat,dc}^{\;k}\right). \end{array}$$

Therefore, the motor primitive weights of the subject, **w_pat_**, are extracted as in:

(4) $${\text{w}}_{pat}^{\;k}={\text{p}}_{\text{h}}{\text{​}}^{†}\tau_{pat,n}^{\;k},$$

where ${\text{p}}_{h}^{†}$ denotes the Moore-Penrose inverse of the motor primitives extracted from the gait used as reference.

The subject weights, **w**_*pat*_, are compared with the reference weights, **w**_*h*_, element-by-element. The relative error between these weights provides a measurement of how distant the subject's gait is from the reference gait. This disparity, Δ**w**, should be addressed by the robotic device during the next swing phase and is denoted simply as:

(5) $$\begin{array}{l} \Delta {\text{w}}^{\;k}={\text{w}}_{h}-{\text{w}}_{pat}^{\;k} \end{array}$$

The difference between the constant offset values is also addressed by the robot. Thus, the torque to be exerted by the robot in the swing phase of the next stride of the right leg, $\tau_{r}^{k+1}$, in given by:

(6) $$\tau_{r}^{\;k+1}=\tau_{h,max}{\text{p}}_{\text{h}}\Delta {\text{w}}^{\;k}+\left(\tau_{h,dc}-\tau_{pat,dc}^{\;k}\right)$$

All quantities related to the subject are denoted by the subscript **pat**, which refers to the word patient. Though no patient was subjected to the test, the variables were named after this word to illustrate the purpose of the control algorithm, which is aimed at people with some sort of motor impairment, e.g., stroke survivors.

To avoid abrupt oscillations, the effective torque to be exerted by the robot, **τ**_*r,e*_, considers the weight deficiency from the last step and the step before the last one. This approach leads to the computation of an exponentially weighted moving average (EWMA). A diagonal matrix, α, composed of α_*i*_ factors, *i* = 1, 2, 3 weights the robot torque as in:

(7) $$\begin{array}{l} \tau_{r,e}^{\;k+1}=\alpha \tau_{r}^{k+1}+\left(\text{I}-\alpha \right)\tau_{r}^{k}. \end{array}$$

During the first step, no robot torque is applied, as there is no previous step. During the second step onward, the robot torque is applied. Exceptionally during the second swing, α is set to unit. Afterward, α, is set to 0.15, a value found empirically.

This robot torque is exerted at the robot joint with the aid of an inner PI torque controller, whose gains were set to *k*_*p*_ = 370 and *k*_*i*_ = 3.5 based on previous works with the robotic device. The robot only acts during the swing phase. During the stance phase transparency is aimed, so the robot torque is set to zero and only the inner torque control loop takes place. The phase detection is hard-coded within the control algorithm and is based on pre-recorded trajectories.

All operations are shown in matrix notation because the control algorithm is proposed to work on three joints of each leg. However, because only the knee joint is actuated, here only this component of the robot torque is used.

The robot exerts torque only when the subject weights are not greater than the reference weights. Otherwise, the robot would restrain the subject's movement, applying an opposing torque. This would lead to a control strategy focused on replicating a reference torque curve, which is not the aim of this work. The same is true for the constant component of the torques when they are greater than the reference values.

**Figure 3:**
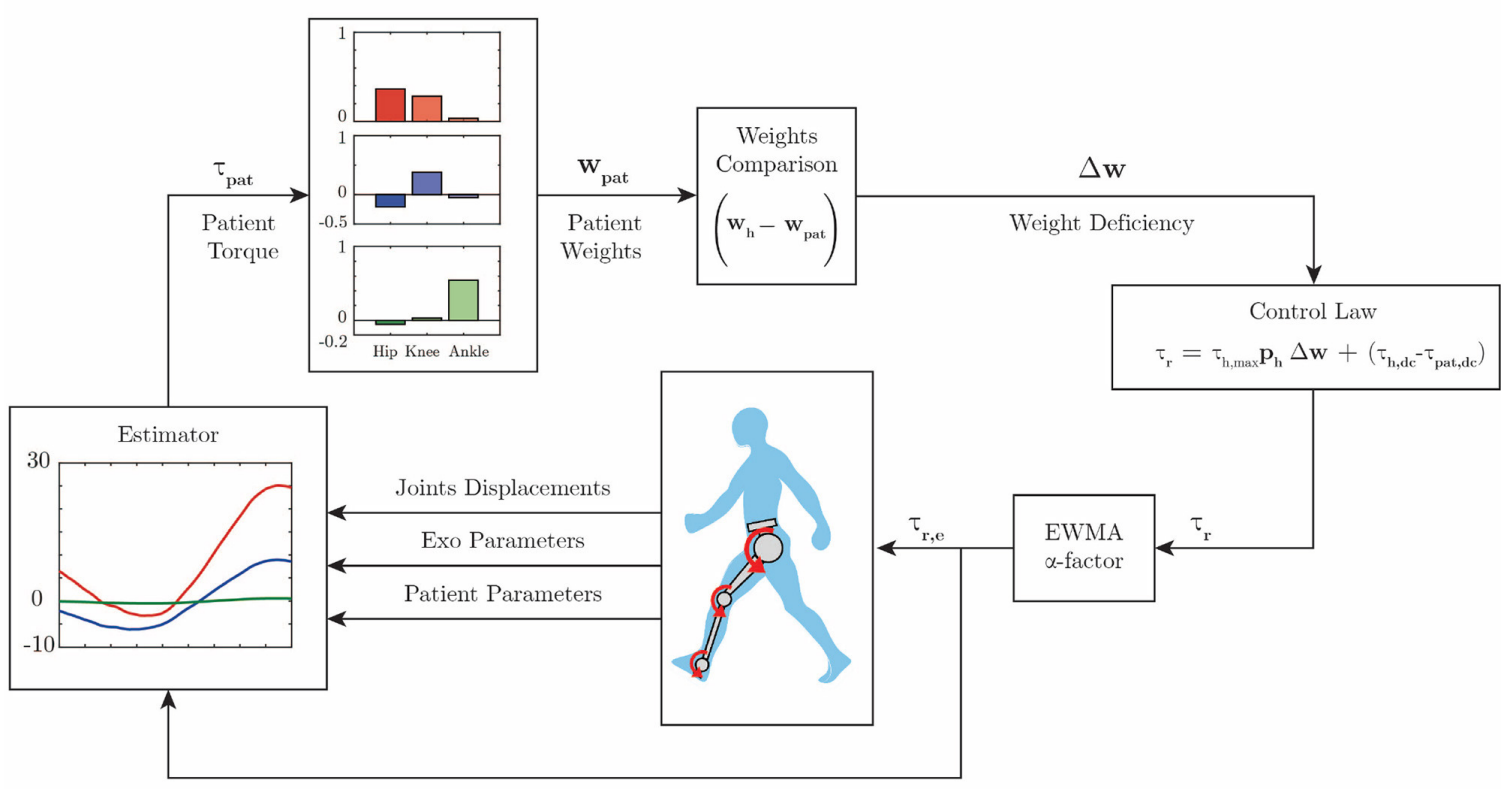
Control diagram. The patient hip (red), knee (blue), and ankle (green) torques are estimated. Further, the motor primitive weights are extracted according to Equation (4). These weights are compared with reference weights, and then Equation (6) computes the assistive robot torque. The robot will exert an exponentially weighted moving average of the computed torque during the next swing phase.

However, when the subject exerts more torque than necessary, but in the opposing direction, which in practice results in opposed-sign weights of greater magnitude than the reference, the robot does act in order to correct the gait. Figure 3 shows the complete block diagram of the proposed adaptive control algorithm.

## 3. Test Protocol

Two tests were performed with 5 healthy subjects (1 female, 4 male, 30 ± 6 years, 73 ± 6 kg, 1.77 ± 0.05 m) in order to evaluate the control algorithm and the motor primitive weights behavior, and one test was performed with one healthy subject (male, 25 years old, 71 kg and 1.76 m) to evaluate the algorithm convergence and adaptability to changes in the mode of walk.

In the first test, the subjects were instructed to walk at 1.5 km/h on a treadmill for 2 min. This yields data for approximately 50 steps. In this test, the subjects walk in an active manner, as if they were without the exoskeleton. The expected robot torque is small, as the subjects produce all the necessary torque to perform the walk, dispensing further assistance. The joints trajectories and torques are expected to match, to a certain extent, the ones obtained during the procedure to extract motor primitives. Further, the behavior of all weights are analyzed.

In the second test, the subjects walked at 1.0 km/h on a treadmill for 2 min. This time, they were instructed to walk slower and also to keep the right leg passive during the swing phase, i.e., to offer no resistance in case the robot tried to move it. Hence, the motor primitive weights relative to the knee joint are expected to result in values lower than the reference, causing the robot to exert an assistive torque over the swing phase. Further, the behavior of all weights are analyzed.

The third test consisted of a combination of the first and the second test and was performed with one healthy subject. He was instructed to walk actively for 30 s at 1.0 km/h, and passively for 30 s at the same speed, completing 1 min of walk. This yields data for 25 steps. Therefore, it could be evaluated whether the control algorithm would adapt to different patient behaviors in real time.

During all tests, the joint displacements, torques, and the motor primitive weights of the subjects were stored for further analysis. The extracted weights of the subject were divided into three categories. The weights could be considered *healthy* weights, which comprised weights with equal or greater magnitude and same direction as the reference weights. In this case, the robot did not actuate. The weights could also be considered *deficient*, which comprised weights below the absolute reference value, regardless of their direction. These weights and their behavior over the first two tests is analyzed. In this case, the robot produced torque during the next swing, in order to assist the subject. Last, the weights could be considered *asynchronous*, which comprised weights with greater magnitude as the reference, but with opposing sign. Although the robot exerted torque in order to correct the subject's gait, these weights are not considered deficient, as their occurrence is rather consequence of a lack of synchrony between the actual gait phase and the phase of the control algorithm, than the consequence of an impaired walk, in which there is a lack of weight to perform the movement.

The primitive extraction procedure and the analysis of data yielded from all tests are processed by means of routines programmed in MATLAB®software (2015a, The MathWorks, Inc., Natick, Massachusetts, United States).

## 4. Results

Herein the experimental results obtained from the motor primitive extraction procedure are shown. These data are further used as reference values within the control algorithm. In the sequence, the results of a set of simulated patient conditions as defined in the previous section are presented (active, passive, and a combination of these two).

### 4.1. Reference Primitive Torques and Weights

**Figure 4:**
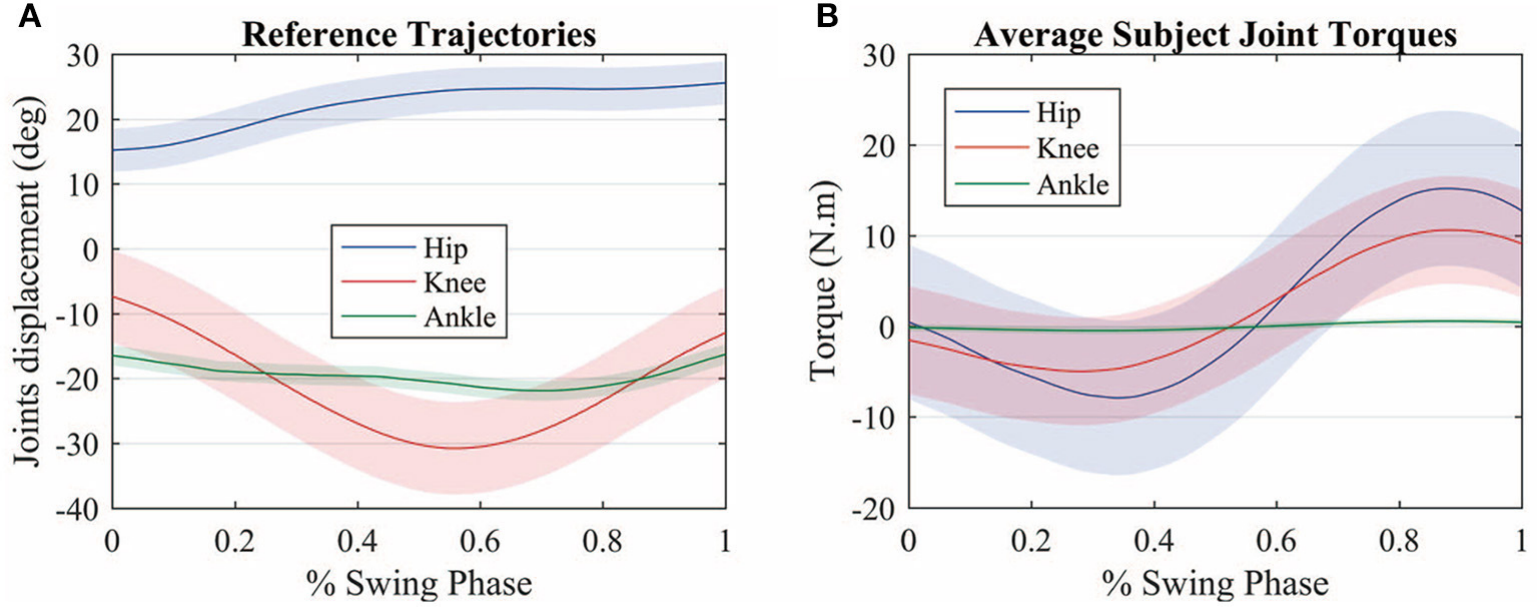
**(A)** Average measured joint displacements (solid lines) during the swing phase. **(B)** Average estimated joint torque profiles (solid lines). The shaded region denotes the standard deviation of the measurements.

Figure 4A shows the average measured joint displacements (solid lines) during the swing phase. Figure 4B shows the average estimated joint torque profiles (solid lines). The shaded region denotes the standard deviation of the measurements.

**Figure 5:**
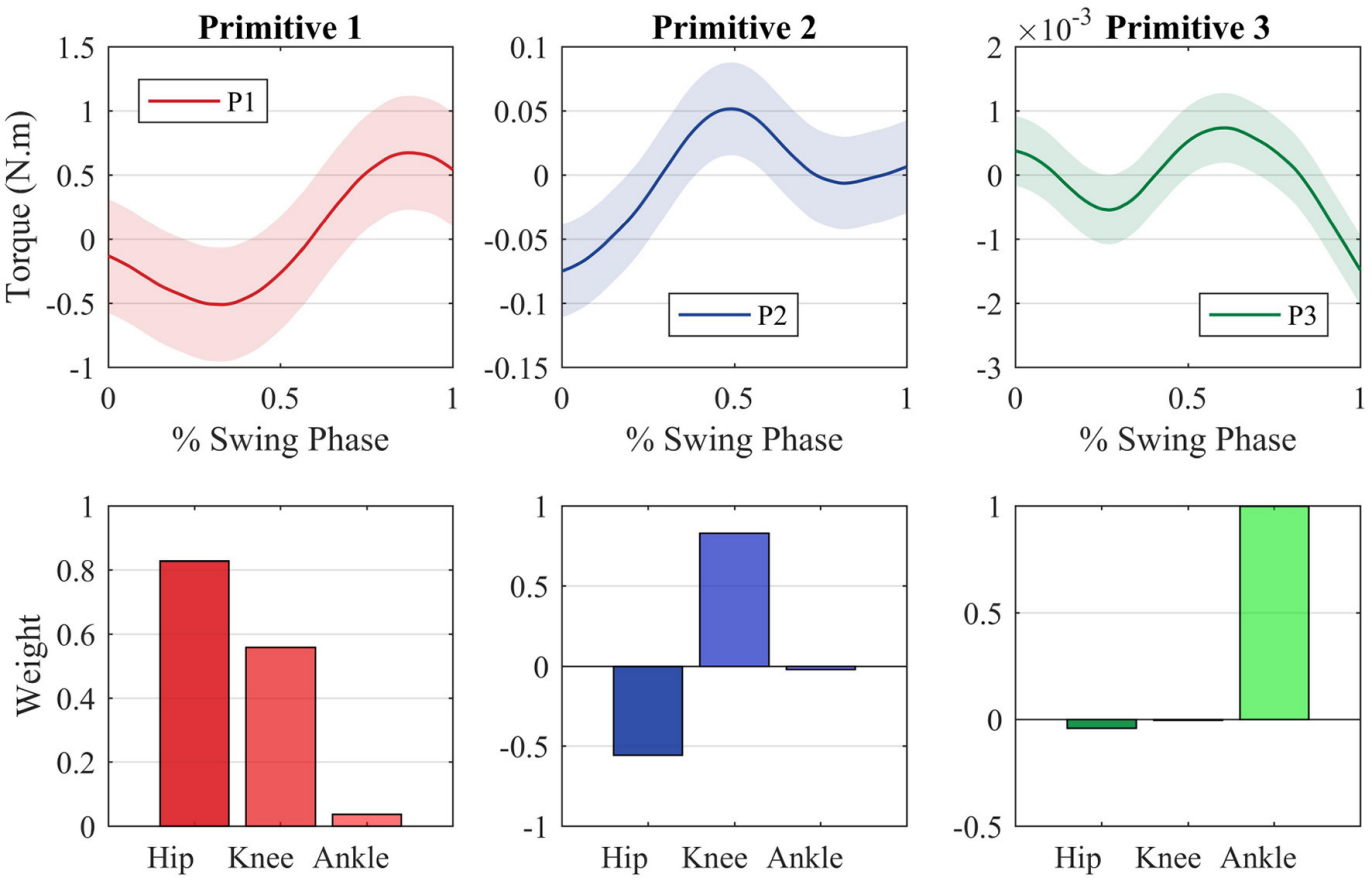
Motor primitives extracted through the PCA method and the motor primitive weights regarding each joint. The joint torques can be reconstructed by making a weighted sum of each primitive with regards to each joint.

Figure 5 shows the average primitive torque profiles from the extraction method described in section 2.2 along with the respective weight regarding each joint. The shaded region denotes the standard deviation of the measurements.

The reference weights employed as reference for the control law are described by the following matrix:

$$\begin{array}{l} {\text{w}}_{h}=\left[\begin{array}{c} {\text{w}}_{h,hip}\\ {\text{w}}_{h,knee}\\ {\text{w}}_{h,ankle} \end{array}\right]=\left[\begin{array}{ccc} 0.8784 & -0.4750 & -0.0539\\ 0.4768 & 0.8786 & 0.0282\\ 0.0340 & -0.0505 & 0.9981 \end{array}\right]\\ \;\pm \left[\begin{array}{ccc} 0.0097 & 0.0142 & 0.0044\\ 0.0140 & 0.0087 & 0.0574\\ 0.0284 & 0.0498 & 0.0015 \end{array}\right]. \end{array}$$

If one multiplies each primitive curve by the respective joint weight and sum all the three components, the average profile torque can be reconstructed for all joints. It is important to notice that each primitive is strictly related to a specific joint. The first primitive, for instance, is tight to the hip joint, which can be noted by the fact that the hip weight of this primitive (0.8784) is greater when compared with the other two weights (0.4768 and 0.0340).

These weights ponder the influence of each primitive on the torque observed at each joint. A negative weight means that the primitive torque must have its direction changed before being multiplied by the weight and being summed to the other primitives that compose the joint torque. The control algorithm will not provide assistance in the case the subject's weights are at least equal to the reference ones. If his weights are greater than the reference, the subject is exerting more torque than necessary. If the weights are greater in magnitude but with an opposing sign, the subject is exerting more torque than necessary and in the wrong direction. In this case, the subject is not considered necessarily in need of assistance to perform the movement, as he is capable of exerting torque of greater or same magnitude as the reference.

It can be noted that during the swing phase the ankle joint exerts almost no torque. This is due the fact that the exoskeleton shoe is rigid to a certain extent, in order to aid post-stroke patients with foot-drop to perform the walk properly. Therefore, the ankle joint can be left out for this application. Hence, the reference weights matrix is further simplified as

$$\begin{array}{l} {\text{w}}_{h}=\left[\begin{array}{c} {\text{w}}_{h,hip}\\ {\text{w}}_{h,knee} \end{array}\right]=\left[\begin{array}{cc} 0.8784 & -0.4750\\ 0.4768 & 0.8786 \end{array}\right]\pm \left[\begin{array}{cc} 0.0097 & 0.0142\\ 0.0140 & 0.0087 \end{array}\right] \end{array}$$

The data extraction also provides an average of the maximum torque, which is the maximum hip torque:

$$\begin{array}{l} \tau_{h,max}=25.8798\text{ Nm}. \end{array}$$

The constant component regarding the average torque of each joint is given by

$$\begin{array}{l} \tau_{dc}=\left[\begin{array}{c} \tau_{dc,hip}\tau_{dc,knee} \end{array}\right]=\left[\begin{array}{cc} 12.4015 & 4.2152 \end{array}\right]\\ \;\pm \left[\begin{array}{cc} 9.7966 & 1.2715 \end{array}\right]\text{ Nm}. \end{array}$$

It can be noted that there is considerable variation among the torque offset values. This is due to the nuances in the gait pattern of the individuals, as well as their characteristics, such as body mass and leg length.

The constant components of the knee and ankle torques are small when compared with the hip component. Moreover, a steadier robot behavior was achieved when these constant values were not considered, since the robot would abruptly raise its torque to this constant value as soon as the swing phase started. Thus, they were not considered for this test, in order to obtain a smoother robot assistance and steadier gait. To extract the patient weights, **w**_*pat*_, and compare with the reference ones, the algorithm must receive the pseudo-inverse of the primitive torques, ${\text{p}}_{h}^{†}$. To reduce computation time, the pseudo-inverse is calculated beforehand and provided to the algorithm. Once the healthy parameters were set, the tests were performed.

### 4.2. Test Results

**Figure 6:**
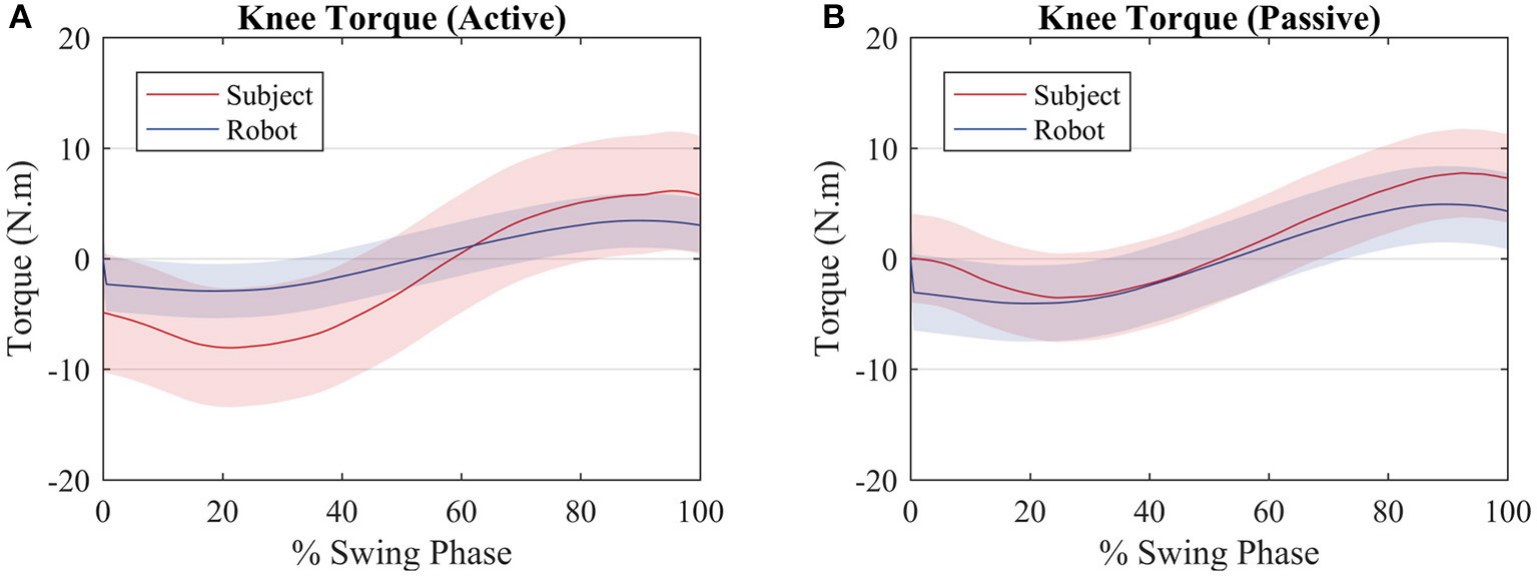
Average torque exerted by the subjects (red) and by the robot (blue) for an **(A)** active walk and a **(B)** passive walk. Shaded regions denote the standard deviation of the measurements.

Though there is not an explicit torque reference value to be tracked by the control algorithm, it is expected that, for the first test, in which the subjects walked actively, the robot exerts less torque than the subject, when compared with the second test, in which the subjects walked passively. In the first case, Figure 6A (active), the robot produces a torque with a RMS value of 2.5 Nm, whereas the user produces a torque with a RMS value of 7.3 Nm, equal to the reference value of 7.3 Nm RMS. In the second case, Figure 6B (passive), the robot produces more torque (RMS = 3.5 Nm) since the user is producing less torque at the knee joint (RMS = 5.6 Nm). It must be noted that it is not possible to prevent the user from exerting torque at the knee joint. When the results of each subject are considered rather than the average among them, for the active walk the minimum RMS torque produced by the robot was 1.2 Nm RMS, when the user was producing a torque of 10.5 Nm RMS. With respect to the passive walk, the maximum torque of the robot was 4 Nm RMS and the subject, 4.4 Nm RMS.

**Figure 7:**
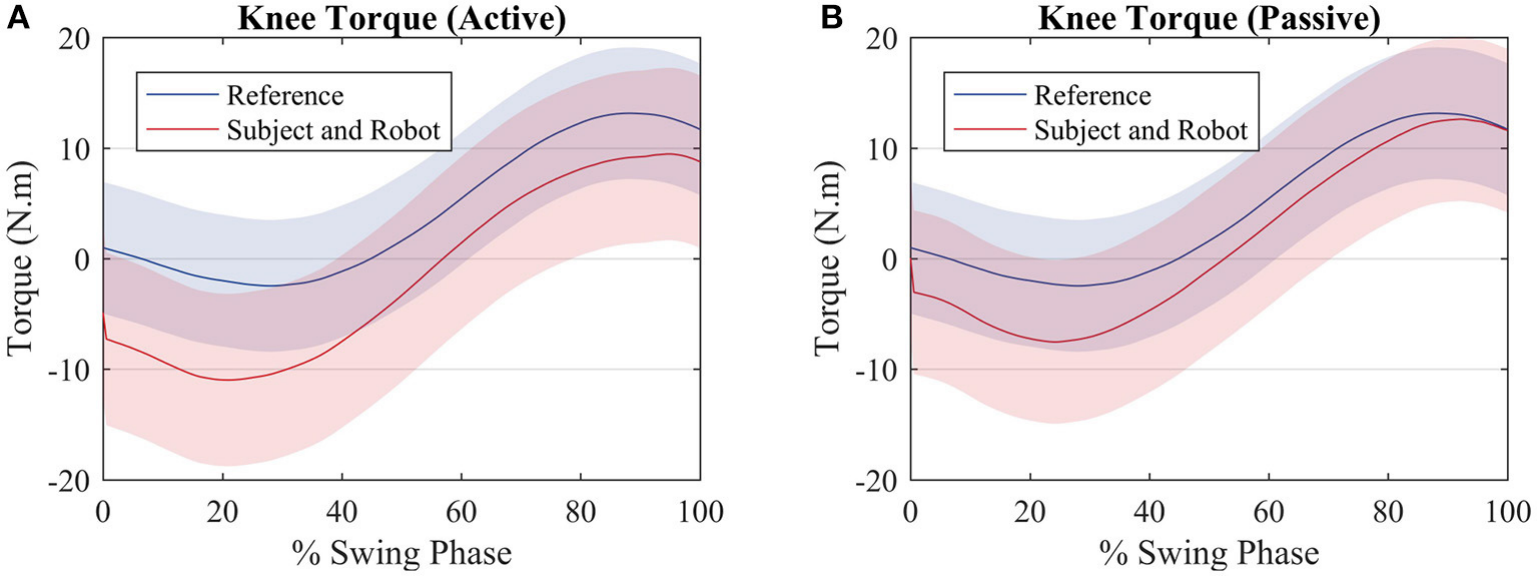
Average torque exerted by the subjects (red) and average torque value used to compute the reference (blue) for an **(A)** active walk and a **(B)** passive walk. Shaded regions denote the standard deviation of the measurements.

When the subject and the robot torque are compared with the reference, it can be noted that for both scenarios the two torques sum up in order to get closer to the reference torque as depicted by Figure 7A (active) and Figure 7B (passive).

**Figure 8:**
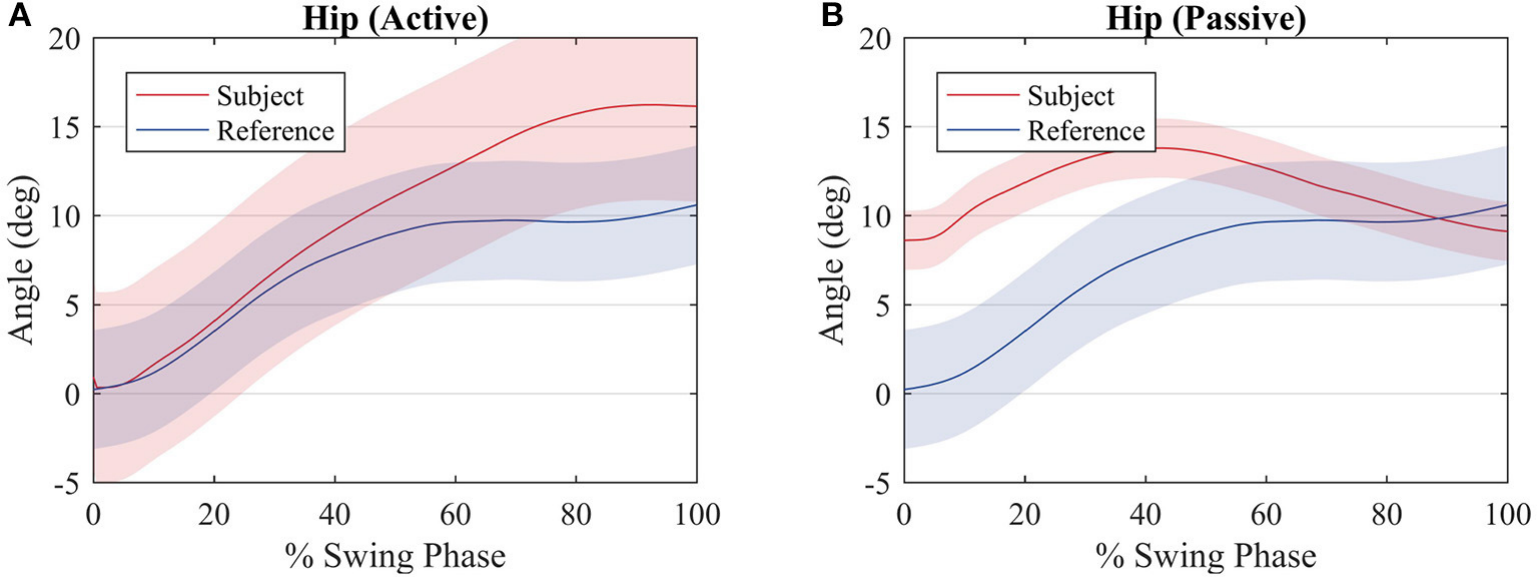
Average hip joint displacement of the subjects (red) and average hip joint displacement during the procedure to compute the reference values (blue) for an **(A)** active walk and a **(B)** passive walk. Shaded regions denote the standard deviation of the measurements.

Figure 8 shows a comparison between the hip joint positions of the subject during the primitive extraction procedure (blue) and during the tests (red). Despite an offset value, which is due to the position of the exoskeleton around the subject's hip, the trajectories are similar among all subjects, and they relate to the reference profile, though, in the active walk, the amplitude of the movement is greater.

**Figure 9:**
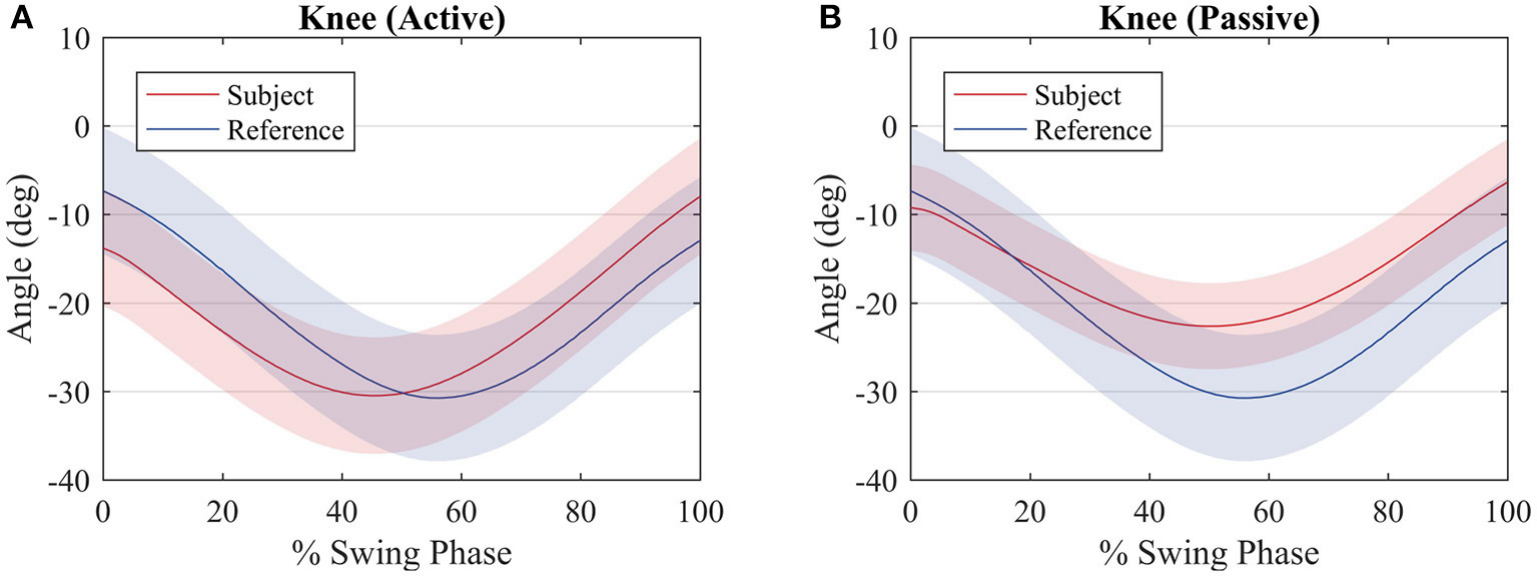
Average knee joint displacement of the subjects (red) and average hip joint displacement during the procedure to compute the reference values (blue) for an **(A)** active walk and a **(B)** passive walk. Shaded regions denote the standard deviation of the measurements.

Figure 9 shows a comparison between the knee joint positions of the subject during the primitive extraction procedure (blue) and during the tests (red). The trajectories are similar among all subjects, and they relate to the reference profile, regardless of the mode of walk, since there is robot actuation at the knee joint. It can be noted that the knee joint is closer to the reference during the passive mode of walk.

**Figure 10:**
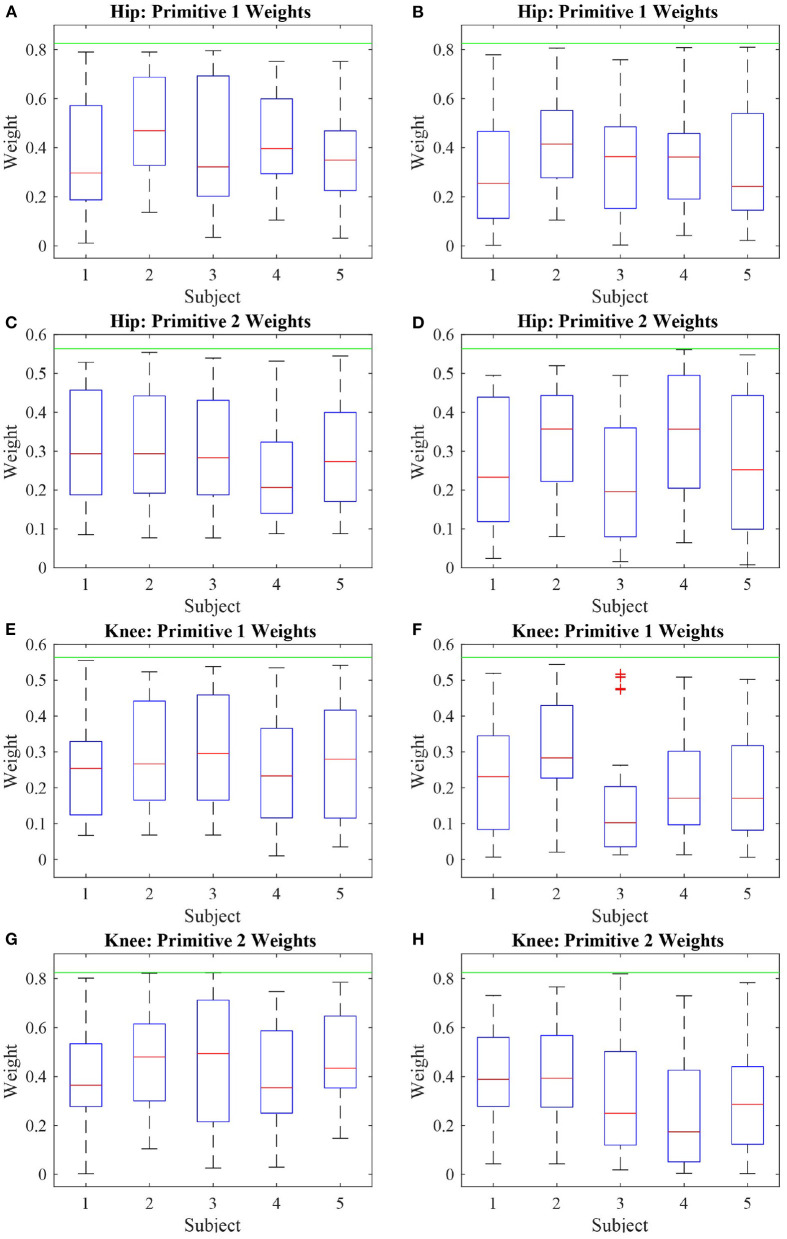
Boxplot of the absolute value of the deficient weights of five subjects performing two modes of walk: active **(A,C,E,G)** and passive **(B,D,F,H)**. It can be noted that the weights with respect to the first primitive are more affected than the weights related to the second primitive, due to the knee actuation and the absence of actuation at the hip joint.

With respect to the motor primitive weights, only the weights that were below the reference value, regardless of their signal, were analyzed, in order to evaluate the influence of the robot actuation between the two modes of walk. To simplify this analysis, these weights were summarized in a boxplot and compared with the other subjects, as depicted in Figure 10.

**Figure 11:**
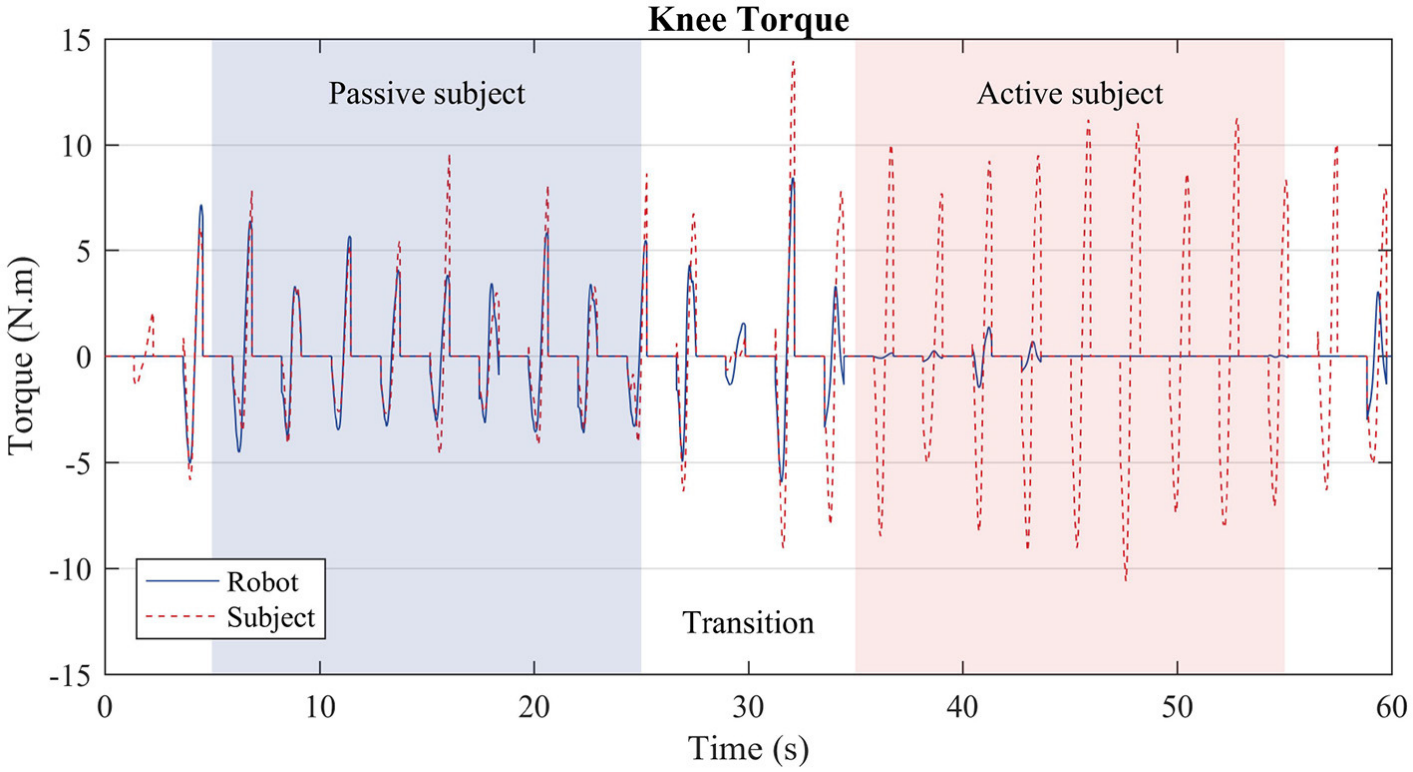
Subject torque (red dashed lines) and robot torque (solid blue line) in the third test. During the first stage (blue region), the user was instructed to walk passively, i.e., the treadmill slowed down, and the subject prevented from moving more than necessary the knee joint of the right leg. During the second stage (red region), the user walked actively, i.e., as if he were not wearing the robot exoskeleton; thus, the robot produced considerably less torque.

The third test evaluated the algorithm performance during gait with oscillating characteristic. Hence, the user walked actively for 30 s and passively for more 30 s. Results are depicted in Figure 11.

## 5. Discussion

As seen in Figure 6, the robot produces less torque during an active mode of walk when compared with the passive mode of walk. However, the robot torque is still present because even though the subjects produce torque when they walk actively, not always their torques have the exact primitive weights as the reference ones. A better clarification on why the robot acted as such is given further through an analysis of the patient weights in Figure 10. Therefore, the robot torque is present so that the sum of the subject and the robot torque gets closer to the reference torque value, as in Figure 7.

The hip joint trajectory is depicted in Figure 8 for an active and passive mode of walk. The trajectory is not tracked so accurately when compared to the knee joint, due to the lack of actuation on this joint. This will reflect on deficient weights with respect to the hip joint and the first motor primitive.

With respect to the knee joint trajectory, it can be noted in Figure 9 that for both modes of walk the trajectory is close to the reference. In fact, for the passive mode of walk, the trajectory is closer when compared with the active mode of walk, due to the fact that most of the movement is performed by the robot. During the active walk, the user walks at a faster pace and the robot, sensing that the knee joint torque of the subject is sufficient or greater to perform the movement, does not oppose to the user movement. This is expected since the robot is supposed to only provide assistance rather than make the user follow strictly a pre-recorded trajectory.

One can notice that no explicity trajectory or torque tracking is included in the control algorithm. Still, torque and trajectory could still be tracked, indirectly, to a certain extent, so that it matched the reference values.

As the hip joint was not actuated, most hip weights were expected to fall within the range of deficiency. However, due to the knee actuation, the weights of the hip regarding the second primitive were kept at steady values of magnitude during most of the walk.

During the active and passive mode of walk, 58% of the hip weights relative to the first primitive were deficient. With respect to the hip weight relative to the second primitive, during the active walk they were deficient 45% of the test, and, in the passive case, 51%. The knee weights with respect to the first primitive were deficient over 40% of the active walk, as 51% for the passive walk. The knee weights with respect to the second primitive were deficient 53% of the active walk, and 63% of the passive walk. Even though the weights with respect to the knee joint were deficient, the trajectory and torque tracking were ensured by the algorithm. The same is not true with respect to the hip joint, as it was not actuated.

Figure 10 shows the behavior of these weights considered deficient. It is important to analyze the deficient weights with more detail because anything below the threshold value established by the reference value is considered deficient. However, due to the antropomorphic characteristics of each person, if the primitive torques are considered to be the same, the weights must vary in order to account for the gait particularities of each person. It can be noted that most weights during the active phase are located above the median (particularly truth for most subjects in Figures 10A,C,E,G) whereas, for the passive walk, most weights are located below the median (Figure 10B) or the median values are lower when compared to the Figure beside it (Figures 10D,F,H).

In the third test the algorithm acted in a way that replicated what was observed in the first and second tests separately. During the transitions, i.e., the white regions of Figure 11, the robot torque is prone to oscillations, as the subject behavior was changing from a passive mode of walk to an active one. Over three to four steps, the robot converges to the expected behavior. During the passive stage (blue region), the robot torque (solid blue line) complements the subject's torque (dashed red line), whereas during the active stage (red region), the robot exerts almost no torque, since the subject is capable of producing the necessary torque by himself.

The hip weights with regards to the first primitives, Figures 10A,B, presented a behavior similar to the weights of the knee joints regarding the same primitive (Figures 10E,F). Likewise, there was similarity between the behavior of the hip and knee weights with relation to the second primitive (Figures 10C,D,G,H). This shows that the joint weights share characteristics with other joint weights with respect to the same primitives rather than with the weights of the same joint. As a consequence, it can be inferred that the first primitive relates to the hip joint the same way the second primitive relates to the knee joint. Therefore, at the same time it seems rather convenient that the actuation over one joint is capable of propagating its effects over the other joints, which could imply that a smaller number of actuators could be used, the results also suggest that the sole actuation of one joint is not capable of compensating for all weight deficiencies pertaining the actuated joint itself.

The results also show that the sole actuation of the knee could guarantee indirectly, through motor primitive analysis, that the reference joint trajectory was tracked to a certain extent. On the other hand, a position control strategy may not guarantee that the motor primitive weights are close to a reference, though it still may result in functional patient trajectories.

Regarding the convergence and adaptability experiment, the results suggest that the algorithm converges within three to four steps to a steady behavior, once the gait characteristics of the subject are analyzed in terms of primitive weights. The robot ceases to actuate once the user produces enough torque, thus sufficient weights, automatically. This ability to adapt guarantees a more comfortable and safe walk, and prevents the scenario in which the subject produces less torque because the robot produces all the necessary torque instead.

The current setup shall be improved in order to extend the results for different modes of walk and before doing tests with impaired individuals. For instance, the actuation of the hip joint is crucial to compensate for weight deficiencies not only at the hip joint, but also at other joints, as concluded with the results exposed here. Extending the number of healthy subjects is important for comparing different motor primitive curves to be used as reference values. The analysis of motor primitives of impaired subjects is also a subject of study, in order to evaluate over therapy how the robotic device assistance affects their primitives. Moreover, a better comprehension of the motor primitives of an impaired subject may lead to better clues on how to approach the weight deficiency problem. Finally, extensive testing with a greater number of healthy subjects is fundamental to later pursue the validation of the control algorithm with impaired subjects, affected either by stroke, SCI or other types of motor impairments which compromise the lower limbs movement.

## 6. Conclusion

This work presents a bio-inspired control algorithm based on motor primitives for a modular lower limbs exoskeleton, in order to aid the rehabilitation of individuals with motor impairment, especially post-stroke victims. First, the motor primitives along with their weights were extracted from healthy subjects. To perform this task, a torque estimation algorithm together with the Principal Component Analysis were employed. Thereafter, tests with healthy subjects wearing the right leg of the exoskeleton were executed to evaluate the controller performance in three scenarios: when the subject walks actively; when the subject shows some torque deficiency, by walking slower than expected; and when there is a combination of these two cases. In all cases, the algorithm evaluates the motor primitive weights of the current subject comparing them with the weights of a healthy subject. When deficiency is observed, the robot properly exerts a complementary torque during the swing phase, to assist the subject to perform this movement. It could be noted a relation between weights of different joints with regards to the same primitive. This shows that even though the sole actuation of one joint has influence on the weights of other joints, it is unable to compensate for all the weight deficiencies of the same joint. Moreover, the results suggest that the knee joint trajectory could be tracked indirectly at some extent solely based on the primitive weights analysis. These results are part of the ongoing effort to develop adaptive control strategies for rehabilitation robots taken into account the specific characteristics and current conditions of a patient. To perform tests with more healthy subjects and with a greater number of actuated joints of the exoskeleton are two crucial steps in order to refine the control algorithm, in order to later perform tests with impaired subjects.

## Data Availability Statement

The original contributions generated for the study are included in the article/supplementary material, further inquiries can be directed to the corresponding author/s.

## Ethics Statement

The studies involving human participants were reviewed and approved by Ethics Committee of the Federal University of São Carlos (Number 26054813.1.0000.5504). The patients/participants provided their written informed consent to participate in this study.

## Author Contributions

PN and IO contributed to the conception and design, acquisition of data, analysis, interpretation of data, and the drafting of the manuscript. AS contributed to the conception, design, interpretation of data, and revision of the manuscript. All authors contributed to the article and approved the submitted version.

## Conflict of Interest

The authors declare that the research was conducted in the absence of any commercial or financial relationships that could be construed as a potential conflict of interest.
